# Decreased Bilateral FDG-PET Uptake and Inter-Hemispheric Connectivity in Multi-Domain Amnestic Mild Cognitive Impairment Patients: A Preliminary Study

**DOI:** 10.3389/fnagi.2018.00161

**Published:** 2018-06-05

**Authors:** Xiao Luo, Kaicheng Li, Qingze Zeng, Peiyu Huang, Yeerfan Jiaerken, Tiantian Qiu, Xiaojun Xu, Jiong Zhou, Jingjing Xu, Minming Zhang

**Affiliations:** ^1^Department of Radiology, The Second Affiliated Hospital of Zhejiang University School of Medicine, Hangzhou, China; ^2^Department of Neurology, The Second Affiliated Hospital of Zhejiang University School of Medicine, Hangzhou, China

**Keywords:** mild cognitive impairment, cerebral metabolism, corpus callosum, resting-state functional MRI, voxel-mirrored homotopic connectivity

## Abstract

**Background:** Amnestic mild cognitive impairment (aMCI) is a heterogeneous condition. Based on clinical symptoms, aMCI could be categorized into single-domain aMCI (SD-aMCI, only memory deficit) and multi-domain aMCI (MD-aMCI, one or more cognitive domain deficit). As core intrinsic functional architecture, inter-hemispheric connectivity maintains many cognitive abilities. However, few studies investigated whether SD-aMCI and MD-aMCI have different inter-hemispheric connectivity pattern.

**Methods:** We evaluated inter-hemispheric connection pattern using fluorine-18 positron emission tomography – fluorodeoxyglucose (^18^F PET-FDG), resting-state functional MRI and structural T1 in 49 controls, 32 SD-aMCI, and 32 MD-aMCI patients. Specifically, we analyzed the 18^F^ PET-FDG (intensity normalized by cerebellar vermis) in a voxel-wise manner. Then, we estimated inter-hemispheric functional and structural connectivity by calculating the voxel-mirrored homotopic connectivity (VMHC) and corpus callosum (CC) subregions volume. Further, we correlated inter-hemispheric indices with the behavioral score and pathological biomarkers.

**Results:** We found that MD-aMCI exhibited more several inter-hemispheric connectivity damages than SD-aMCI. Specifically, MD-aMCI displayed hypometabolism in the bilateral middle temporal gyrus (MTG), inferior parietal lobe, and left precuneus (PCu) (*p* < 0.001, corrected). Correspondingly, MD-aMCI showed decreased VMHC in MTG, PCu, calcarine gyrus, and postcentral gyrus, as well as smaller mid-posterior CC than the SD-aMCI and controls (*p* < 0.05, corrected). Contrary to MD-aMCI, there were no neuroimaging indices with significant differences between SD-aMCI and controls, except reduced hypometabolism in bilateral MTG. Within aMCI patients, hypometabolism and reduced inter-hemispheric connectivity correlated with worse executive ability. Moreover, hypometabolism indices correlated to increased amyloid deposition.

**Conclusion:** In conclusion, patients with MD-aMCI exhibited the more severe deficit in inter-hemispheric communication than SD-aMCI. This long-range connectivity deficit may contribute to cognitive profiles and potentially serve as a biomarker to estimate disease progression of aMCI patients.

## Introduction

The amnestic mild cognitive impairment (aMCI) represents an intermediate stage between normal aging and Alzheimer’s disease (AD) (Petersen et al., 2014). According to impaired cognitive domains number, aMCI patients could be further categorized into two subtypes: single-domain aMCI (SD-aMCI), characterized by relatively selective memory impairment and the multi-domain aMCI (MD-aMCI), indicating extensive deficits involving at least one other domain (Winblad et al., 2004; Busse et al., 2006). Previous epidemiology study shows that MD-aMCI has a higher risk of clinical progression than the SD-aMCI (Golob et al., 2007). Therefore, to investigate the mechanism underlying these aMCI subtypes may efficiently facilitate clinical early intervention and management. Recently, neuroimaging studies pointed out that the MD-aMCI displays more diffuse gray matter atrophy (Haller et al., 2010; Zhang et al., 2012) and lower brain activity than the SD-aMCI, mainly involving default mode network (DMN) and frontoparietal regions (Li et al., 2014). Despite these studies shedding light into the aMCI pathological mechanism to some extent, it remains unclear whether SD-aMCI and MD-aMCI have different inter-hemispheric connection pattern.

Compared to the other neuroimaging indices, insufficient attention paid to the direct inter-hemispheric connectivity of aMCI patients. Anatomically, the connectivity between hemispheres is a core mode of the brain intrinsic functional architecture. Moreover, this bi-hemispheric communication procedure substantially affects many cognitive domains, including executive and memory functions (Salvador et al., 2008; Stark et al., 2008; Saar-Ashkenazy et al., 2016). Until now, only two functional MRI studies directly explored the inter-hemispheric functional connectivity in aMCI patients. However, two studies drew the entirely different conclusion. Specifically, Wang et al. (2015) demonstrated that MCI patients exhibited an enhanced inter-hemispheric functional connectivity in the sensorimotor cortex to resist the cognitive decline; however, another work reported the negative result between aMCI and controls (Qiu et al., 2016). Given that aMCI is a heterogeneous condition, we thus hypothesized that this inconsistency might attribute to the different damage pattern of inter-hemispheric connectivity between SD-aMCI and MD-aMCI patients.

Notably, some previous findings focusing on corpus callosum (CC) supported this hypothesis. Anatomically, CC acts as the most robust commissural white matter bundle to maintain the functional connectivity between the hemispheres (Roland et al., 2017). Specifically, some studies demonstrated that the AD and MCI patients exhibit the CC shape change, atrophy, and impaired diffusivity indices impairment, especially in the posterior part (Janowsky et al., 1996; Di Paola et al., 2010; Ardekani et al., 2014; Rasero et al., 2017). Subsequently, some studies further demonstrated patients with MD-aMCI, but not SD-aMCI, have reduced mean diffusivity (MD) in the whole CC compared to controls; moreover, decrease of MD in the CC body is associated with decreased general cognition and executive ability (Li et al., 2013). On the other hand, as a valid diagnostic biomarker in dementia studies, fluorine-18 fluorodeoxyglucose (^18^F FDG) positron emission tomography (PET) research also shows more diffuse hypometabolism in MD-aMCI than the SD-aMCI. Interestingly, these hypometabolism regions are mostly located in the bilateral homotopic precuneus (PCu) and the temporoparietal cortex (Caffarra et al., 2008; Cerami et al., 2015). Considering the evidence of CC degeneration and bilateral homotopic hypometabolism, we inferred that different inter-hemispheric connectivity damage pattern might exist between aMCI subgroups.

To test this hypothesis, we analyzed the structural T1 image, resting-state functional MRI (rsfMRI), ^18^F PET-FDG, neuropsychological scales, and pathological biomarkers in a relatively large aMCI sample from ADNI database. Firstly, we analyzed inter-hemispheric homotopic functional connectivity by using a voxel-wise method, namely voxel-mirrored homotopic connectivity (VMHC) (Zuo et al., 2010; Luo et al., 2018b). The potential inter-hemispheric structural connectivity was evaluated by estimating CC volume. We divided CC into five part due to its different anatomical connection [i.e., genus connects the frontal areas, while body and splenium part connects the temporal and parietal areas (Fischl, 2012)]. Moreover, to test whether regions with inter-hemispheric disruption accompanied by metabolic abnormalities, we also analyzed ^18^F FDG-PET data in a voxel-wise manner. Additionally, we also correlated neuroimaging indices with cognition and pathological biomarkers (reflecting by CSF and amyloid imaging). Our study aims to (i) compare the alteration of the inter-hemispheric connectivity and the metabolism between aMCI subtypes; (ii) explore the possible interactions between different neuroimaging modalities; and (iii) explore the relationships between neuroimaging indices and cognition.

## Materials and Methods

### Alzheimer’s Disease Neuroimaging Initiative

Data used in this study were obtained from the Alzheimer’s disease Neuroimaging Initiative (ADNI) database ^1^. The ADNI was launched in 2003 by the National Institute on Aging (NIA), the National Institute of Biomedical Imaging and Bioengineering (NIBIB), the Food and Drug Administration (FDA), private pharmaceutical companies, and non-profit organizations, as a $60 million, 5-year public–private partnership. The primary goal of ADNI has been to test whether serial magnetic resonance imaging (MRI), PET, other biological markers, and clinical and neuropsychological assessment can be combined to measure the progression of mild cognitive impairment (MCI) and early Alzheimer’s disease (AD). Determination of sensitive and specific markers of very early AD progression is intended to aid researchers and clinicians in developing new treatments and monitor their effectiveness, as well as lessen the time and cost of clinical trials.

### Subjects

All procedures performed in studies involving human participants were following the ethical standards of the institutional and national research committee and with the 1975 Helsinki Declaration and its later amendments or comparable ethical standards. The ADNI project was approved by the Institutional Review Boards of all participating institutions, and all participants at each site signed informed consent. Based on ADNI GO and ADNI 2 database, we identified 49 healthy right-handed subjects (normal controls, NC) and 64 aMCI patients, who had undergone structural scans, rsfMRI scans, FDG-PET scans, and neuropsychological evaluation. We downloaded the study data from the ADNI publicly available database before April 15, 2017. According to ADNI protocol, to be classified as healthy controls, the Mini-Mental State Examination (MMSE) for the subject should be between 24 and 30 (inclusive), and clinical dementia rating (CDR) score should be 0. Besides, the subject has no signs of depression (Geriatric Depression Scale, GDS <6) or possible dementia. To be defined as aMCI, the subject had an MMSE score between 24 and 30 (inclusive), memory complaint, objective abnormal memory evidence, and a CDR score of 0.5. Besides, aMCI patients’ general cognition preserved well (cannot meet AD diagnostic criteria). Also, there were no signs of depression (GDS score <6) in aMCI patients.

Followed by exclusion standards: the subject who has a history of apparent head trauma, other neurological or major psychiatric disorder, and alcohol/drug abuse. Additionally, subjects were also excluded if they exhibited a significant vascular disease risk history, defined as Hachinski Ischemia Scale (HIS) scores higher than 4.

### Neuropsychological Evaluation and MCI Subtypes Diagnosis

All subjects underwent comprehensive neuropsychological tests. Content includes general mental status (MMSE score, Alzheimer’s Disease Assessment Scale, ADAS) and other cognitive domain, including memory function (Auditory Verbal Learning Test, AVLT; Wechsler Memory Scale-Logical Memory, WMS-LM, including immediate and delayed score), processing speed (Trail-Making Test, Part A, TMT-A), visuospatial function (Clock-Drawing Test, CDT), executive function (Trail-Making Test, Part B, TMT-B), and language ability (Boston Naming Test, BNT). According to the previous studies, we used the composite scores for executive functioning and memory (Crane et al., 2012; Gibbons et al., 2012).

The aMCI patients were divided into two subtypes by the performance in cognitive domains (Shu et al., 2012). Specifically, SD-aMCI refers to aMCI patients having an impairment in memory alone; MD-aMCI refers to aMCI patients having an impairment in memory and other cognitive domain (at least one). We defined impairment as the presence of a test scoring 1.5 standard deviations (SD) below the average score, from age- and education-matched healthy controls from ADNI. Specifically, the total number of healthy controls from ADNI is 198, mean age is 73.03 ± 6.20, and mean education attainment is 16.47 ± 2.45. No significant difference existed in terms of age and education level (*p* > 0.05) between ADNI controls and aMCI subjects (mean age: 73.33 ± 5.69; education level: 15.84 ± 2.45). Finally, we enrolled 49 out of 198 ADNI controls who undergone structural scans, rsfMRI scans, and neuropsychological evaluation for subsequent analyses. Meanwhile, 32 MD-aMCI and 32 SD-aMCI patients entered the subsequent analyses.

### CSF Data

We downloaded the CSF dataset from the ADNI database. Based on CSF samples, Aβ_1-42_, total tau (t-tau) and phosphorylated tau (p-tau_181_) were measured (Bittner et al., 2016). These CSF biomarkers, including Aβ_1-42_, t-tau, and p-tau_181_, are also useful candidates as they are intimately related to amyloid plaques, neuronal death, and accumulation of tangles, respectively (Jovicich et al., 2016). Moreover, the ratio of p-tau/Aβ_1-42_ was also calculated due to its association with cognition and disease progression (Landau et al., 2010). Notably, not all subjects in the present study had the CSF samples due to the invasive procedure of lumbar puncture. Thus, the final CSF sample for analyses included 26 out of the 49 NC, 30 out of the 32 SD-aMCI, and 28 out of the 32 MD-aMCI patients.

### Amyloid PET Data

Given that amyloid PET shows more potent than CSF markers for MCI prognosis, we further downloaded the results of ^18^F-florbetapir PET data from the ADNI database, namely UCBERKELEYAV45_11_14_17.csv (Bouallègue et al., 2017). The detailed processing procedure was described previously (Landau et al., 2013). In the following analysis, we used the results of composite SUVR value (intensity-normalized by a whole cerebellum region).

### Image Acquisition

The 3.0-Tesla Philips MRI scanner was used to scan all participants. Based on MPRAGE T1-weighted sequence, structural images were acquired with the following parameters: repetition time (TR) = 2300 ms; echo time (TE) = 2.98 ms; inversion time (TI) = 900 ms; 170 sagittal slices; within plane FOV = 256 × 240 mm^2^; voxel size = 1.1 × 1.1 × 1.2 mm^3^; flip angle = 9°; and bandwidth = 240 Hz/pix. Based on echo-planar imaging sequence, rsfMRI images were acquired with the following parameters: time points = 140; TR = 3000 ms; TE = 30 ms; flip angle = 80^°^; the number of slices = 48; slice thickness = 3.3 mm; spatial resolution = 3.31 × 3.31 × 3.31 mm^3^; and matrix = 64 × 64. Meanwhile, PET images were obtained by either a 30-min six frame scan acquired 30–60-min post-injection or a static 30-min single-frame scan acquired 30–60-min post-injection.

### Image Preprocessing

The image data were preprocessed by using the Data Processing Assistant for rsfMRI, DPASF^2^, based on the Statistical Parametric Mapping (SPM 12)^3^ and rsfMRI Data Analysis Toolkit, REST^4^. Briefly, we removed the first 10 image volumes of rsfMRI scans were for the signal equilibrium and the subjects’ adaptation to the machine noise. The remaining 130 image volumes were corrected for both timing differences and head motion (24 Friston; Friston et al., 1996). We excluded the data with more than 2.0 mm maximum displacement in any directions (*X-, Y-*, and *Z*-axis) and 2.0 degrees of any angular motion. Subsequently, the T1-weighted images were co-registered to the mean rsfMRI image based on a thorough rigid-body transformation and normalized to the Montreal Neurological Institute (MNI) space, subsequent resliced into of 3 mm × 3 mm × 3 mm cubic voxels. Besides, we performed linear trends and temporal filter (0.01 Hz < *f* < 0.08 Hz). To remove any motion residual effects and other non-neuronal factors, we corrected covariates (including six head motion parameters, WM signal, CSF) in the following functional connectivity analysis. After preprocessing, we obtained each subject’s 4D residual functional volume in native functional space. These 4D data were registered to the MNI152 space with 2 mm resolution (affine transformation). Due to the dispute of removing the global signal, we omit to regress out the global signal. Given the effect of the micro-motion artifact, we evaluated the frame-wise displacement (FD) value for each subject. Subjects were screened and excluded if FD value >0.5 mm on more than 35 volumes.

### Voxel-Mirrored Homotopic Connectivity Analysis

For VMHC computation, firstly, the mean T1 image was generated from the average of 113 spatially normalized T1 images. Then, we flipped the left hemisphere along the midline of the *x*-axis to obtain the symmetric brain template, further, to create the final template. Subsequently, the each subject’s T1 image was co-registered nonlinearly to the customized symmetric template. The same deformation field subsequently applied to the rsfMRI. More details regarding VMHC data processing are available in the literature (Anderson et al., 2010; Zuo et al., 2010). Based on Pearson’s correlation, the homotopic RSFC between any pair of symmetrical inter-hemispheric voxels was estimated, then transformed to Fisher’s *Z* map. Finally, we defined these resultant values as the VMHC and used it in following analyses.

### Corpus Callosum Volume

We evaluated the CC volume and its subregions based on FreeSurfer software package (Version 5.1^5^) as described previously (Fischl, 2012; Reuter et al., 2012). In detail, FreeSurfer automatically segmented CC into five sections, with each section representing a fifth of the total area. We defined five segments as anterior (CC-A), mid-anterior (CC-MA), central (CC-C), mid-posterior (CC-MP), and posterior CC part (CC-P).

### FDG-PET Voxel-Wise Analysis

We downloaded the ^18^F FDG-PET data from the ADNI database in their most processed formats (PET Pre-processing protocol^6^). Specifically, pre-processed scans were generated following co-registration dynamic (to the first acquired frame), averaging frames, spatial re-orientation (AC–PC line), intensity normalization (within subject-specific mask), and smoothing (uniform isotropic spatial resolution 8 mm full width at half maximum kernel). The subsequent processing procedures of FDG-PET were conducted by combining T1 MRI images, which were similar to VMHC processing mentioned above. Firstly, each FDG-PET scan was co-registered to the individual’s corresponding T1 MRI image in native space. Then, these structural scans were segmented, and the resulting GM partitions were spatially normalized using the SPM 12. The spatial normalization parameters (deformation fields) that had been estimated from the gray matter segment normalization were subsequently applied to the co-registered FDG-PET images so that both the GM, VMHC, and the FDG-PET images were in MNI space. To remove inter-individual nuisance variability in tracer metabolism, we intensity-normalized FDG-PET image via dividing it by the average FDG-PET value of the reference region (cerebellar vermis, defined by the AAL regions within the MNI atlas, manually checked by experienced radiologists, MMZ). Finally, we created the standardized uptake value ratio (SUVR) images for the following analysis.

### Statistics

All statistical analyses were performed using IBM SPSS statistical software. The TMT-A, TMT-B, and ADAS-cog were log-transformed because of a positively skewed distribution. We used ANOVA to detect group differences in terms of age, education level, cognitive ability, and AD-related pathological results. We used the *post hoc* pairwise *t*-tests if ANOVA was significant (*p* < 0.05, corrected by Bonferroni).

Regarding VMHC results, the ANCOVA was used to explore different brain regions among the three groups, with controlling for age, education level, and gender. For the objective to explore, the threshold set at height *p* < 0.05 and cluster level *p* < 0.05 (Gaussian random field, GRF corrected). We performed the *post hoc* pairwise *t*-tests within the brain regions identified by the ANCOVA (*p* < 0.005 Bonferroni corrected). Regarding the FDG-PET SUVR image, we performed ANCOVA to explore metabolic differences, with controlling for age, education level, and gender [*p* < 0.001 at height and *p* < 0.05 at the cluster level (GRF corrected)]. Similarly, we performed the *post hoc* pairwise *t*-tests if ANCOVA was significant (*p* < 0.005, Bonferroni corrected). Also, we calculated the between-group CC subregions volume difference by using ANCOVA corrected by age, education level, gender, and total intracranial volume (TIV). We performed *post hoc* pairwise *t*-test if ANOVA was significant (*p* < 0.05, corrected by the least significant difference, LSD).

Further, we correlated imaging measures to cognitive abilities and also explored the possible interactions between neuroimaging modalities. To achieve the best visual effect, we overlapped between-group difference results of VMHC and FDG-PET SUVR image by the same statistical standard (ANCOVA, corrected by age, education level, and gender, *p* < 0.05 at height, *p* < 0.05 at the cluster level, GRF corrected). Meanwhile, to explore the possible interaction between CC subregions and other modalities, we performed voxel-wise regression analysis (*p* < 0.05 at height, *p* < 0.05 at the cluster level, GRF corrected). Notably, we calculated correlations limited to those indices having significant between-group differences.

## Results

### Patient Characteristics

We displayed quantitative variables as the mean and its SD, and the categorical variable as absolute and relative frequency. **Table 1** shows the demographic characteristics, cognitive abilities, and pathological biomarkers for each group. There were no significant differences in terms of age, education level, and gender composition among the three groups. Regarding the general cognitive ability, we found that three groups exhibited the significant differences in the score of MMSE and ADAS-cog, with MD-aMCI displaying the worst performance. Regarding other cognitive domains, group effects were significant, with the best performance in NC and also the worst performance in MD-aMCI patients.

**Table 1:** Comparison of demographic information, behavioral data, and pathological biomarkers.

	NC	SD-aMCI	MD-aMCI	*F* (χ^2^)	*P*-value
Number	49	32	32		
Age	73.33 ± 4.60	72.43 ± 4.25	74.91 ± 5.27	2.29	0.11
Female	31	15	15	3.00	0.22
Education	16.24 ± 2.60	16.47 ± 2.24	15.25 ± 2.65	2.20	0.12
**General mental status**					
MMSE	29.02 ± 1.20	28.34 ± 1.68	27.16 ± 1.71	15.03	<0.001^bc^
Log-transformed ADAS	0.91 ± 0.17	1.07 ± 0.19	1.21 ± 0.20	24.21	<0.001^abc^
**Spatial processing**					
CDT	4.76 ± 0.48	4.75 ± 0.44	3.78 ± 1.18	19.69	<0.001^bc^
**Language**					
BNT	28.57 ± 1.44	28.09 ± 2.07	27.09 ± 11.36	0.55	0.58
**Memory**					
WMS-LM immediate	14.73 ± 2.76	9.88 ± 3.17	7.88 ± 2.83	60.49	<0.001^abc^
WMS-LM delayed	13.96 ± 2.99	7.97 ± 2.78	5.75 ± 3.06	84.93	<0.001^abc^
AVLT-Total	43.22 ± 9.01	37.34 ± 9.63	31.25 ± 8.78	16.85	<0.001^abc^
**Processing speed**					
Log-transformed TMTA	1.51 ± 0.12	1.47 ± 0.08	1.66 ± 0.15	23.82	<0.001^bc^
**Executive function**					
Log-transformed TMTB	1.86 ± 0.16	1.86 ± 0.13	2.18 ± 0.16	51.75	<0.001^bc^
Composite memory	1.05 ± 0.51	0.44 ± 0.59	0.06 ± 0.61	31.99	<0.001^abc^
Composite executive	0.80 ± 0.65	0.87 ± 0.51	–0.35 ± 0.51	47.82	<0.001^bc^
**Pathological results**					
Aβ_1-42_ (pg/ml)	1222.19 ± 552.35	1132.06 ± 616.57	963.00 ± 438.94	1.85	0.16
t-tau (pg/ml)	248.52 ± 84.62	275.76 ± 90.51	300.33 ± 134.34	2.18	0.12
P-tau_181_ (pg/ml)	23.43 ± 9.46	25.69 ± 9.30	29.34 ± 15.01	2.30	0.11
p-tau/Aβ_1-42_	0.03 ± 0.02	0.03 ± 0.02	0.04 ± 0.02	2.83	0.06
Composite PET-PiB	1.14 ± 0.19	1.20 ± 0.20	1.29 ± 0.25	4.54	0.01^b^

*Data are presented as means ± SD. Notably, the mean levels of Aβ_*1*-*42*_, t-tau, and p-tau_*181*_ in **Table 1** only represent the subjects who had CSF samples. ^*a*^*Post hoc* paired comparisons showed significant group differences between NC and SD-aMCI, after Bonferroni correction (*p* < 0.05). ^*b*^*Post hoc* paired comparisons showed significant group differences between NC and MD-aMCI, after Bonferroni correction (*p* < 0.05). ^*c*^*Post hoc* paired comparisons showed significant group differences between SD-aMCI and MD-aMCI, after Bonferroni correction (*p* < 0.05). MMSE, Mini-Mental State Examination; ADAS, the Alzheimer’s Disease Assessment Scale-Cognitive Subscale; WMS-LM, Wechsler Memory Scale-Logical Memory; TMT, Trail-Making Test; CSF, cerebrospinal fluid; CDT, Clock-Drawing Test; AVLT, Auditory Verbal Learning Test; BNT, Boston Naming Test.*

Regarding AD-related pathological biomarkers, MD-aMCI patients exhibited a significantly higher composite PET PiB SUVR value than NC (*p* < 0.01). Meanwhile, although MD-aMCI group presented a trend of the increased level of t-tau and p-tau_181_ as well as decreased Aβ_1-42_, no significant differences existed among groups (*p* > 0.05). Also, we also failed to detect the difference in the p-tau_181_/Aβ_1-42_ ratio among groups.

### Glucose Metabolism Differences

**FIGURE 1:**
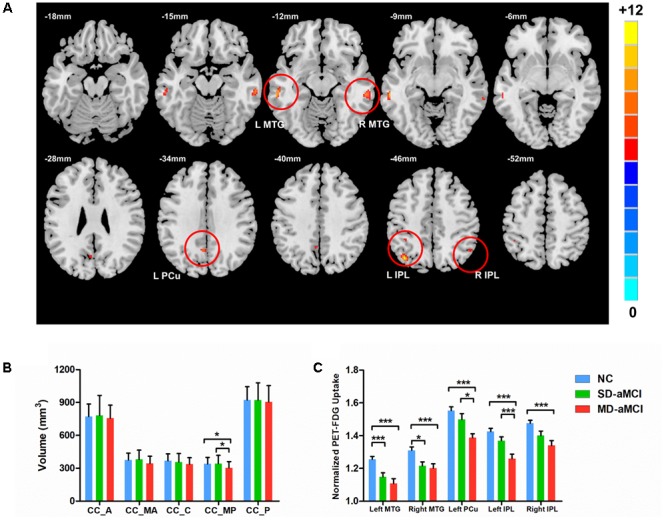
Upper **(A)** shows the voxel-wise comparison result: brain areas with the significant difference of metabolism (normalized by cerebellum vermis) among NC, single-domain aMCI (SD-aMCI), and multi-domain aMCI (MD-aMCI), by controlling for age, education level, and gender. The threshold set *p* < 0.001 at height and *p* < 0.05 at the cluster level, GFR corrected. Lower figures show the histogram for MD-aMCI (red), SD-aMCI (green), and NCs (blue). Error bars represent SD. ^∗^ represents *p* < 0.05 while ^∗∗∗^ represents *p* < 0.001, corrected for multiple comparisons. **(B)** The *post hoc* analyses were performed within the areas identified by the ANCOVA (*p* < 0.05). The MD-aMCI patients displayed the significantly decreased volume of the CC-MP compared to the other two groups. **(C)** We performed the *post hoc* analyses within the areas identified by the ANCOVA (*p* < 0.005). Among the three groups, the MD-aMCI patients exhibited the lowest metabolism in bilateral MTG, bilateral inferior parietal lobe (IPL), and left precuneus (L-PCu). Other abbreviation: A, anterior; MA, mid-anterior; C, central; MP, mid-posterior; and P, posterior.

Three groups demonstrated the significant metabolic differences in the following regions: namely bilateral middle temporal gyrus (MTG), bilateral inferior parietal lobe (IPL), and left PCu. Subsequent pairwise group comparisons carried out on these clusters revealed that both aMCI patients exhibited a lower metabolism in bilateral MTG. Also, the MD-aMCI demonstrated a reduced metabolism in left PCu and bilateral IPL compared to the SD-aMCI and NC (**Figure 1** and **Table 2**).

**Table 2:** Brain areas with the significant difference of VMHC and PET-FDG among NCs, SD-aMCI, and MD-aMCI patients.

Regions	MNI coordinates			Cluster voxels	Peak intensity
	*X*	*Y*	*Z*		
**VMHC**
Middle temporal gyrus	±51	–54	–3	128	8.30
Precuneus	±3	–57	60	133	7.60
Postcentral gyrus	±60	–21	24	317	8.86
Calcarine gyrus	±3	–75	9	195	9.60
**PET-FDG**
L middle temporal gyrus	–60	–32	–10	59	11.08
	–44	–50	12	14	8.48
R middle temporal gyrus	62	–28	–14	52	9.37
L precuneus	–6	–54	38	38	8.86
L inferior parietal lobe	–38	–68	48	60	11.61
	–38	–42	48	24	9.46
R inferior parietal lobe	48	–58	44	13	8.60

*Regarding VMHC results, the ANCOVA was used to explore differential brain regions among groups [controlling for age, education level, and gender, *p* < 0.05 at height and *p* < 0.05 at the cluster level, Gaussian random field (GRF) corrected]. Regarding the FDG-PET standardized uptake value ratio (SUVR) image, the ANCOVA was used to explore differential brain regions among the three groups (controlling for age, education level, and gender, *p* < 0.001 at height and *p* < 0.05 at cluster level, GRF corrected). The *X, Y*, and *Z* coordinates of the primary peak locations in the MNI space. MD-aMCI, multiple-domain amnestic mild cognitive impairment; SD-aMCI, single-domain amnestic mild cognitive impairment; MNI, Montreal Neurological Institute.*

### Inter-Hemispheric Connection Differences

**FIGURE 2:**
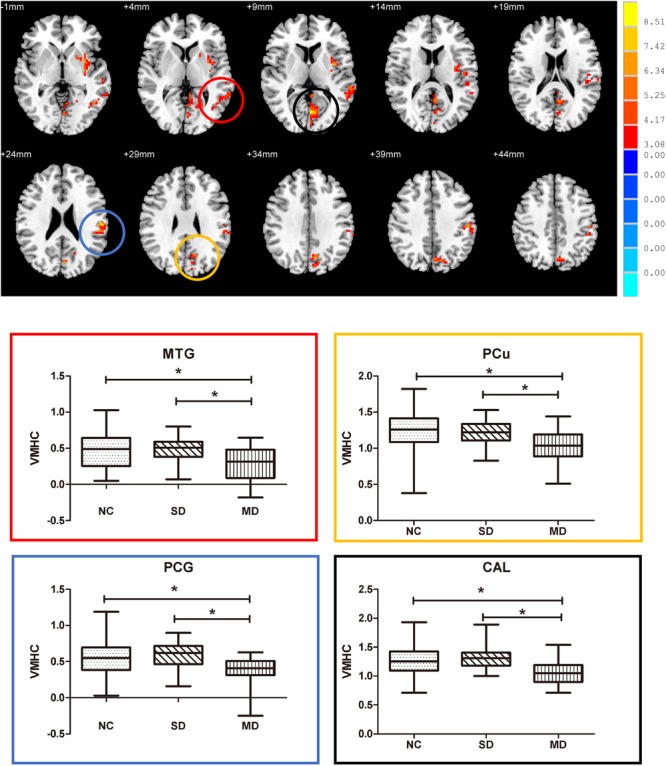
Upper: brain areas with the significant difference of voxel-mirrored homotopic connectivity (VMHC) among normal control (NC), single-domain aMCI (SD-aMCI), and multi-domain aMCI (MD-aMCI), by controlling for age, education level, and gender. The threshold set *p* < 0.05 at height and cluster *p* < 0.05 at the cluster level, GFR corrected. Lower: we performed the *post hoc* analyses within the areas identified by the ANCOVA (corrected by Bonferroni). MD-aMCI patients displayed significantly decreased inter-hemispheric functional connectivity in bilateral middle temporal gyrus, precuneus (PCu), postcentral gyrus (PCG), and CG compared to NC and SD-aMCI patients (*p* < 0.005). But no significant difference existed between SD-aMCI patients and NC. Box graph displays mean VMHC value for middle temporal gyrus (MTG) (red), PCu (yellow), PCG (blue), and CG (black). ^∗^*p* < 0.005, Bonferroni corrected.

Among the three groups, we established the significant VMHC difference in these regions: namely middle MTG, PCu, postcentral gyrus (PCG), and calcarine gyrus (CG). As expected, subsequent pair-wise group comparisons suggested that MD-aMCI exhibited a decreased VMHC in MTG, CG, PCu, and PCG relative to SD-aMCI and NC (*p* < 0.005, corrected by Bonferroni); however, there were no significant differences of VMHC between SD-aMCI and NC (**Figure 2** and **Table 3**).

**Table 3:** Comparisons of VMHC, FDG-PET SUVR, and corpus callosum subregions volume among groups.

	NC	SD-aMCI	MD-aMCI	*F*-value	*P*-value
**Bi-hemispheric functional connectivity**				
Middle temporal gyrus	0.48 ± 0.26	0.48 ± 0.17	0.28 ± 0.23	8.93	<0.001^bc^
Precuneus	1.23 ± 0.26	1.21 ± 0.17	1.02 ± 0.24	10.92	<0.001^bc^
Postcentral gyrus	0.54 ± 0.22	0.60 ± 0.18	0.37 ± 0.20	12.86	<0.001^bc^
Calcarine gyrus	1.25 ± 0.24	1.33 ± 0.20	1.06 ± 0.20	8.71	<0.001^bc^
**Cerebral metabolism**				
L Middle temporal gyrus	1.26 ± 0.12	1.14 ± 0.14	1.11 ± 0.16	11.72	<0.001^ab^
R Middle temporal gyrus	1.31 ± 0.14	1.22 ± 0.14	1.20 ± 0.15	6.94	<0.001^ab^
L Precuneus	1.55 ± 0.15	1.50 ± 0.20	1.39 ± 0.13	9.50	<0.001^bc^
L Inferior parietal lobe	1.42 ± 0.13	1.37 ± 0.13	1.26 ± 0.16	13.32	<0.001^bc^
R Inferior parietal lobe	1.48 ± 0.12	1.40 ± 0.16	1.34 ± 0.17	8.05	<0.001^b^
**CC subregions volume**				
CC-A	771.08 ± 113.95	781.06 ± 183.62	756.16 ± 120.71	0.26	0.77
CC-AP	374.65 ± 62.76	380.88 ± 87.48	343.75 ± 65.36	2.59	0.08
CC-C	367.82 ± 63.85	356.59 ± 77.61	337.59 ± 59.22	1.98	0.14
CC-MP	338.27 ± 61.17	342.06 ± 75.19	303.84 ± 56.78	3.61	0.03^∗^
CC-P	921.65 ± 121.69	921.34 ± 157.03	904.50 ± 149.93	0.17	0.85

*^*a*^*Post hoc* paired comparisons showed significant group differences between NC and SD-aMCI, after Bonferroni correction (*p* < 0.05). ^*b*^*Post hoc* paired comparisons showed significant group differences between NC and MD-aMCI, after Bonferroni correction (*p* < 0.05). ^*c*^*Post hoc* paired comparisons showed significant group differences between SD-aMCI and MD-aMCI, after Bonferroni correction (*p* < 0.05). ^∗^*Post hoc* paired comparisons showed significant group differences between NC and MD-aMCI as well as SD-aMCI and MD-aMCI, corrected by the LSD (*p* < 0.05). Data are presented as means ± SD. CC, corpus callosum; A, anterior; MA, mid-anterior; C, central; MP, mid-posterior; P, posterior.*

We observed the between-group effect on CC-MP subregion (**Figure 1**). Then, the *post hoc t*-test revealed that MD-aMCI exhibited the smallest CC-MP volume among groups, and no significant volume difference existed between SD-aMCI and NC (*p* < 0.05, corrected by LSD).

### Neuroimaging Indices Correlate Cognitive Abilities and Pathological Biomarkers

**FIGURE 3:**
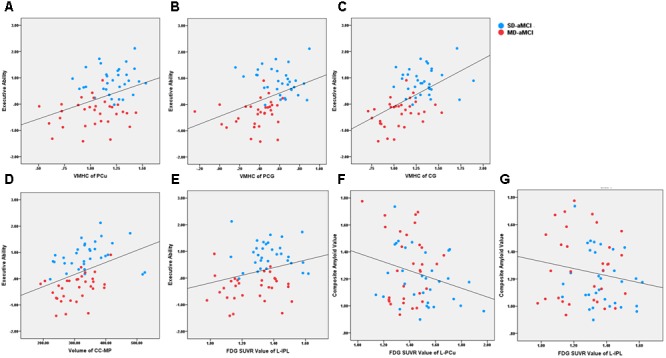
It shows the scatter plot association between neuroimaging indices and behavioral/pathological data across aMCI patients. The blue and red dot represents the SD-aMCI and MD-aMCI patients, respectively. **(A–C)** Regarding functional connectivity, reduced inter-hemispheric functional connectivity in PCu (*r* = 0.39, *p* < 0.001), PCG (*r* = 0.40, *p* < 0.001), and CG (*r* = 0.54, *p* < 0.001) were related to the executive composite score. **(D)** The CC-MP volume was related to the executive composite score (*r* = 0.40, *p* < 0.001). **(E)** The PET-FDG SUVR uptake value in the left IPL (*r* = 0.27, *p* < 0.05) was related to executive function. **(F, G)** The PET-FDG SUVR uptake value in left PCu (*r* = –0.25, *p* < 0.05) and left IPL (*r* = –0.25, *p* < 0.05) were related to composite amyloid value reflecting by ^18^F-florbetapir PET. Abbreviation: aMCI, amnestic mild cognitive impairment; PCu, precuneus; ITG, inferior temporal gyrus; AG, angular gyrus; CG, calcarine gyrus; PCG, postcentral gyrus; IPL, inferior parietal lobe; CC-MP, mid-posterior corpus callosum.

All the correlation analyses we performed were within aMCI patients (**Figure 3**). We observed that reduced left IPL metabolism (*r* = 0.27) was related to worse executive ability (*p* < 0.05). In addition, regarding functional connectivity, we identified that reduced inter-hemispheric connection in PCu (*r* = 0.39), PCG (*r* = 0.40), and CG (*r* = 0.54) were related to worse composite score of executive functioning (*p* < 0.001). Also, the CC-MP volume was related to the composite score of executive functioning (*r* = 0.40, *p* < 0.001).

Regarding AD-related pathological biomarkers, we analyzed the correlation only within the indices having the between-group difference. Our results revealed that hypometabolism of left PCu (*r* = -0.25, *p* < 0.05) and right IPL (*r* = -0.25, *p* < 0.05) was related to the increased amyloid accumulation reflecting composite ^18^F-florbetapir PET value.

### Interaction Between Modalities

As expected, we noted that VMHC and PET-FDG SUVR between-group difference results overlapped well. Specifically, these overlapping included bilateral the MTG, the PCu, the IPL, and the PCG (Supplementary Material 1). Within these overlapping regions, we further observed that VMHC value related to SUVR uptake value within aMCI patient groups (*r* = 0.27, *p* < 0.05).

Within the aMCI patients, the mid-posterior CC volume was related to the bilateral MTG, PCu, CG, and insula (*p* < 0.05 at height and *p* < 0.05 at the cluster level, GRF-corrected Supplementary Material 2). However, we found no significance between mid-posterior CC volume and FDG SUVR uptake value (*p* > 0.05).

## Discussion

This study provides evidence that the MD-aMCI displayed simultaneous decreased bilateral metabolism and reduced inter-hemispheric functional connectivity in MTG, PCu, and parietal regions, accompanying with mid-posterior CC atrophy. However, except hypometabolism in bilateral MTG, no other differences existed between SD-aMCI and NC. Subsequently, the correlation analyses showed that these neuroimaging indices related to executive function across aMCI patients. Moreover, hypometabolism indices related to increased amyloid deposition. In summary, compared to SD-aMCI, the MD-aMCI exhibited the more severe deficit in inter-hemispheric communication. This long-range connectivity deficit may further contribute to cognitive abilities deficits and potentially serve as a biomarker to monitor the disease progression in aMCI patients.

Firstly, our findings suggested that both aMCI patients displayed hypometabolism in bilateral MTG, which is a region involving memory-related network (Ward et al., 2014; Munoz-Lopez et al., 2015). These deficits thus may contribute to the memory deficit in these patients. However, only the MD-aMCI simultaneously displayed inter-hemispheric functional connectivity decrease in MTG, supporting the notion that the MD-aMCI is a more advanced disease form than the SD-aMCI. Notably, one study pointed out that SD-aMCI displayed decreased regional activity in right MTG relative to NC (Luo et al., 2018a). Accordingly, we speculated that the intact inter-hemispheric connectivity in SD-aMCI might compensate its unilateral MTL impairment to some extent. Our neuropsychological results could support this interpretation, showing that MD-aMCI exhibited worse memory performance than SD-aMCI. Besides, other support evidence comes result from ^18^F-florbetapir PET indicating that only the MD-aMCI has increased amyloid deposition than NC. Meanwhile, our findings validated previous studies, which reported that only MD-aMCI patients have cortical thinning, gray matter atrophy, and white matter tract deficit in MTG regions (Seo et al., 2007; Whitwell et al., 2007).

Many evidence suggests that amyloid accumulation preferentially starts in PCu, especially in the left side (Janke et al., 2001; Braak et al., 2006; Palmqvist et al., 2017). As expected, our results showed the MD-aMCI, but not the SD-aMCI, exhibited hypometabolism (left side) and reduced inter-hemispheric functional connection in PCu regions simultaneously. Moreover, we found that the reduced PCu inter-hemispheric functional connection associated with the worse executive function across aMCI patients. This relationship agrees with clinical manifestation in MD-aMCI patients, manifested by more than one cognitive domain impairment. Meanwhile, previous work also documented that MD-aMCI displayed impaired microstructure in PCu (Li et al., 2013). Specifically, by utilizing rsfMRI, one study demonstrated that the MD-aMCI exhibited lower regional activity in PCu than the SD-aMCI and NC. Coincidentally, their work also found that reduced PCu activity was related to worse executive ability (Li et al., 2014). As a central role in the primary executive networks, studies of the AD and MCI frequently reported that regional PCu deficit associated with the decreased executive ability (Sridharan et al., 2008; Chen et al., 2013; Luo et al., 2017a,b). Furthermore, we found that left PCu hypometabolism was related to amyloid deposition. Consequently, we speculated that amyloid-related neurotoxicity in unilateral PCu region might lead to the inter-hemispheric connectivity damage in corresponding areas.

Regarding the parietal cortex, the MD-aMCI exhibited hypometabolism in bilateral IPL and reduced inter-hemispheric PCG functional connectivity; additionally, we found that IPL hypometabolism was related to increased amyloid deposition. Therefore, we again interpreted that the hypometabolism in bilateral IPL may attribute to synaptic dysfunction caused by amyloid (Palop and Mucke, 2010; Tönnies and Trushina, 2017). Moreover, we observed that parietal cortex hypometabolism and inter-hemispheric dysfunction associated with worse executive ability. This relationship could attribute to the reason that both the IPL and PCG have extensive connections with the frontal region (i.e., frontoparietal circuit). Functionally, this circuit could send rich sensory information for processing speed and affect executive ability (Sullivan et al., 2001; Buckner et al., 2008; Luo et al., 2017b). Furthermore, some structural MRI studies support our results. One VBM study pointed out that patients with MD-aMCI exhibited GM reduction and increased mean diffusion in PCG (Li et al., 2013). Meanwhile, using diffusion spectrum imaging (DSI), Chang et al. (2015) highlighted that the MD-aMCI groups have more impairment in the inferior cingulum bundle than the SD-aMCI groups, which anatomically connected with the parietal cortex.

Interestingly, MD-aMCI patients displayed reduced inter-hemispheric RSFC in CG, which is part of the extra-striate visual network and involved with the perception created by visual stimuli. Similarly, one rsfMRI study also illustrated that the MD-aMCI displayed decreased brain activity here (Li et al., 2014). On the other hand, most previous aMCI studies (without dividing into SD-aMCI and MD-aMCI) concluded that aMCI patients displayed increased intrinsic activity in CG (Han et al., 2011; Liu et al., 2012; Lou et al., 2016). This inconsistency may attribute to the aMCI heterogeneity. Besides, we found that decreased inter-hemispheric functional connectivity between bilateral CG was related to worse executive ability. Conceptually, the reaction time includes not only visual processing but also the time required for response execution (Thorpe et al., 1996). Moreover, optical encoding impairment could contribute to the reduction of processing speed (Brébion et al., 2015). Consequently, we interpreted this correlation as that the MD-aMCI patients were difficult to receive the information flows from visual sensors, further, exerting a negative influence on executive ability.

As the dominant white matter pathways, the CC links cortical hubs of the left and right hemispheres together. Here, we observed that MD-aMCI exhibited a smaller mid-posterior CC volume than the SD-aMCI and NC. Moreover, we found the selective mid-posterior CC degeneration in MD-aMCI corresponded well with the fMRI results discussed above. Specifically, regression analysis results showed that mid-posterior CC was significantly related to VMHC results, involving the bilateral MTG, PCu, CG, and PCG. In accord with our results, anatomical evidence showed that the bilateral MTG and PCu are strongly connected through the posterior CC (Cavanna and Trimble, 2006; van der Knaap and van der Ham, 2011; Wang et al., 2014). Additionally, we noted that smaller mid-posterior CC volume was related to worse executive function. This relationship suggests that CC degeneration might contribute to or even accelerate cognitive deficit (Agosta et al., 2015; Farrar et al., 2017; Luo et al., 2017b). Combined with the results of bilateral hypometabolism and inter-hemispheric disconnection, we hypothesized that mid-posterior CC atrophy may reflect the Wallerian degeneration secondary to neuronal loss caused by amyloid deposition (Bozzali et al., 2002).

Without the histological data, it was difficult to describe the exact mechanism underlying inter-hemispheric connectivity deficits in MD-aMCI patients. There are two reasons that may interpret the possible mechanism. Firstly, these deficits may result from the amyloid-related pathological process. Specifically, we found that the between-group difference results of PET-FDG and VMHC overlapped well, including the bilateral MTG, PCu, IPL, and PCG regions. Further, within these overlapping regions, the level of hypometabolism was significantly related to inter-hemispheric decrease. Nevertheless, only the metabolism indices were related to amyloid deposition. We, therefore, speculated that accumulated amyloid plaques in MD-aMCI patients might lead to synapse loss and hypometabolism, further, result in functional connectivity disruption with its contralateral regions (Tönnies and Trushina, 2017). Second, decreased inter-hemispheric functional connectivity may also result from the widespread white matter integrity disruption (White et al., 2009; Luo et al., 2016). Conclusively, these interpretations should be taken with caution due to the lack of animal study and diffusion tensor imaging (DTI) method, respectively.

Some limitations existed in our study. First, this cross-sectional study was failed to assess the long-term effects of hypometabolism and inter-hemispheric disconnection in the subsequent disease progression. Therefore, longitudinal studies are needed to explore the inter-hemispheric connection pattern following AD continuum, from healthy aging to clinical stages. Second, in accord with the inter-hemispheric functional connectivity results, correspondingly, MD-aMCI patients demonstrated selective mid-posterior CC degeneration. However, CC volume only identified the main inter-hemispheric connective tracts, rather than illustrating the precise definition of “inter-hemispheric structural connection.” Therefore, future DTI studies by using tractography or network analysis are needed to provide more detailed information about inter-hemispheric white matter pathways in aMCI patients (Cavanna and Trimble, 2006; Rasero et al., 2017; Tucholka et al., 2018). In the current study, based on the ADNI 2 database, we noticed that subjects do not have both the DTI and the rsfMRI data. Finally, future studies with larger sample sizes are urgently required.

## Conclusion

By using multi-modal neuroimaging methods, this study initially explored the mechanism underlying inter-hemispheric connection pattern in SD-aMCI and MD-aMCI patients. Firstly, our results support the notion that aMCI is heterogeneous. Specifically, MD-aMCI displayed more severe inter-hemispheric communication impairment while the SD-aMCI relatively preserved it. Moreover, our results demonstrate that different inter-hemispheric connectivity damage pattern contributes to distinct clinical symptoms in these two aMCI subtypes. Finally, our study shows that inter-hemispheric connectivity may serve as a potential biomarker to monitor disease progression in aMCI patients.

## Author Contributions

XL study design, analysis, interpretation, and writing. KL, QZ, and TQ analysis and interpretation of data, study concept, and design. PH, XX, MZ, YJ, JX, and JZ manuscript revision and statistical analysis.

## Conflict of Interest Statement

The authors declare that the research was conducted in the absence of any commercial or financial relationships that could be construed as a potential conflict of interest.

## Footnotes

1 adni.loni.usc.edu

2 http://www.rfmri.org/DPARSF

3 http://www.fil.ion.ucl.ac.uk/spm/

4 http://www.resting-fmri.sourceforge.net/

5 http://surfer.nmr.mgh.harvard.edu/

6 http://adni.loni.usc.edu/methods/pet-analysis/pre-processing/
