# Supplementary material for: Decreased Bilateral FDG-PET Uptake and Inter-Hemispheric Connectivity in Multi-Domain Amnestic Mild Cognitive Impairment Patients: A Preliminary Study
Source: Front Aging Neurosci. 2018 Jun 5;10:161. doi: 10.3389/fnagi.2018.00161 (PMC5996941; doi:10.3389/fnagi.2018.00161)

Supplementary Material 1

To achieve the best visual effect, we overlapped between-group difference results of VMHC and FDG PET SUVR image by the same statistical standard (ANCOVA, corrected by age, education, and gender, $p<0.05$ at height, $p<0.05$ at the cluster level, GRF corrected). As the below figure showed, the red and green color represents the regions showing VMHC and PET FDG difference between groups respectively. Notably, the yellow color represents the overlapping region. These overlapping area included bilateral MTG, PCu, IPL, and PCG. After extracting the value of the intersection regions, we found VMHC value was significantly related to corresponding PET FDG SUVR value ($r=0.27$, $p<0.05$; red and blue dot represents MD-aMCI and SD-aMCI patients respectively).

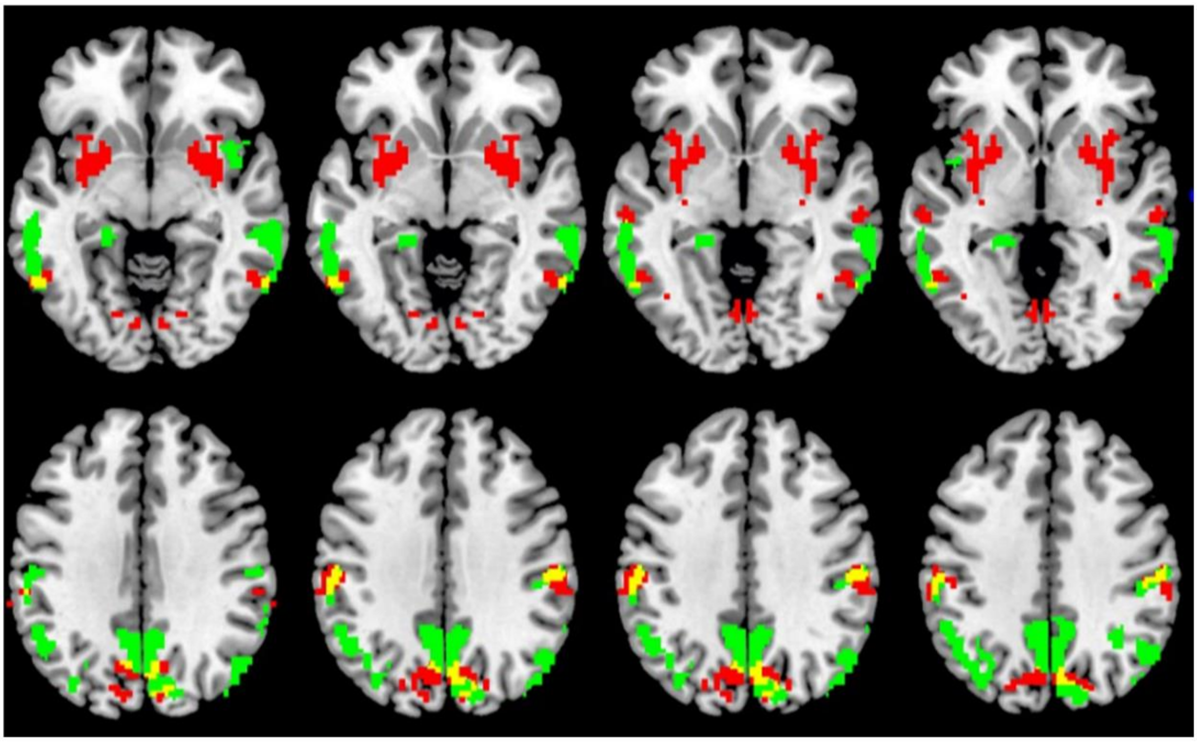

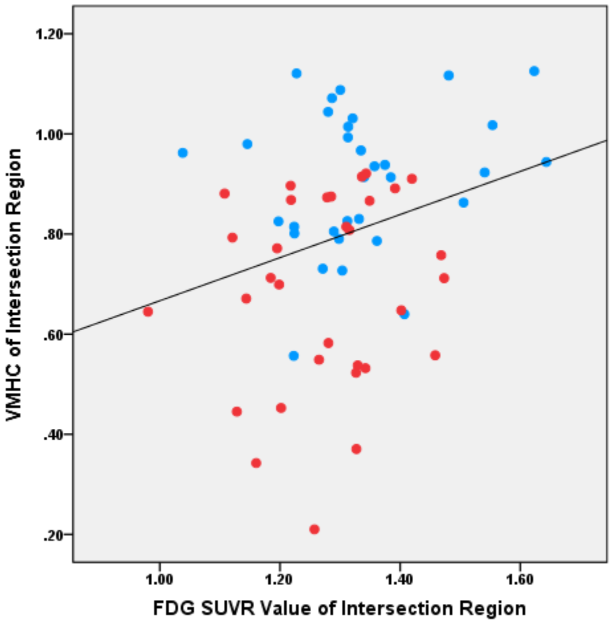

Supplementary Material 2

To explore the possible interaction between CC subregions and other modalities (VMHC and PET FDG), we performed voxel-wise regression analysis ($p<0.05$ at height, $p<0.05$ at the cluster level, GRF corrected). Within aMCI patients, the regression analyses revealed that mid-posterior CC volume was related to inter-hemispheric functional connectivity, including bilateral MTG, PCu, calcarine gyrus and insula (as the figure below). However, we found no significant between mid-posterior CC volume and FDG SUVR uptake value ($p>0.05$).

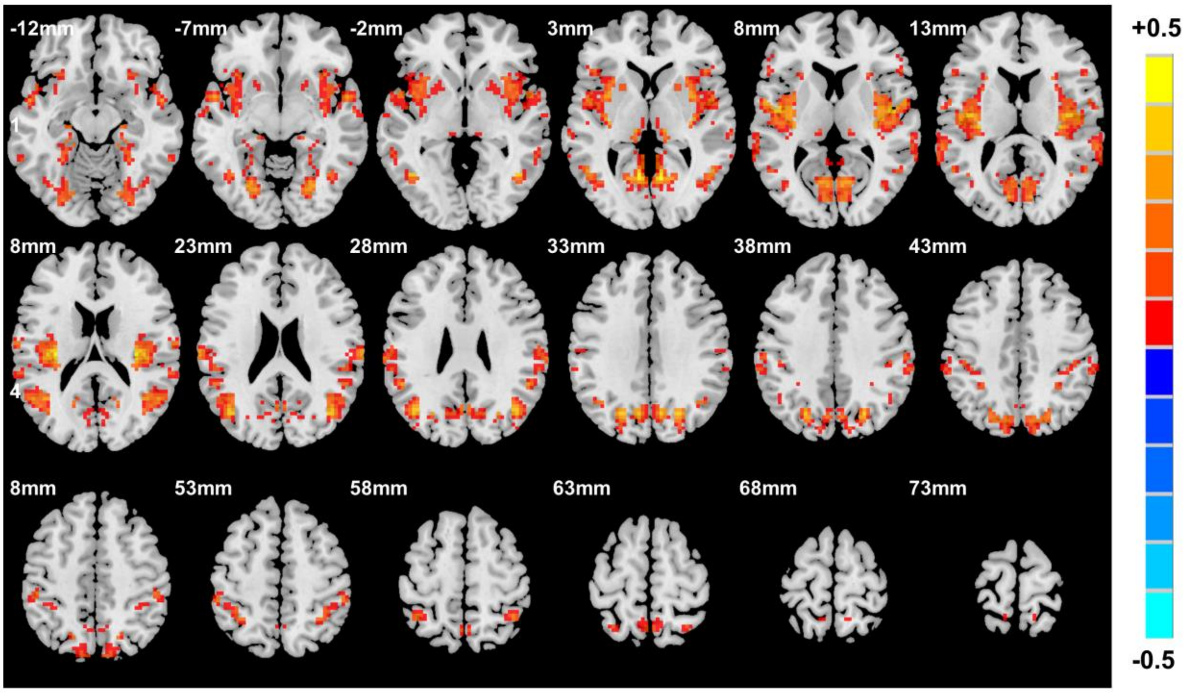

Supplement: Supplementary file 1 [file Presentation_1.PDF]
